# Is access to crisis teams associated with changes in behavioral health mortality?

**DOI:** 10.1093/haschl/qxaf003

**Published:** 2025-01-15

**Authors:** Helen Newton, Tamara Beetham, Susan H Busch

**Affiliations:** Department of Family Medicine, University of North Carolina-Chapel Hill, Chapel Hill, NC 27514, United States; Department of Health Policy and Management, Yale School of Public Health, New Haven, CT 06510, United States; Department of Health Policy and Management, Yale School of Public Health, New Haven, CT 06510, United States

**Keywords:** crisis intervention, behavioral health treatment, suicide, drug overdose, acute alcohol injury

## Abstract

Behavioral health–related mortality—deaths from suicide, drug overdose, and acute alcohol injury—are leading causes of death among US adults. Crisis teams, trained behavioral health professionals who serve as first responders to assess and stabilize clients in crisis, as well as refer to treatment as necessary, have been shown to reduce psychiatric hospitalizations, but whether crisis teams reduce behavioral health mortality has not been studied. We assessed the association between changes in access to crisis team programs and changes in county-level suicide, drug overdose, and acute alcohol injury mortality from 2014 through 2019. We found that 250 (9%) of counties experienced crisis team program entry and another 237 (9%) experienced crisis team program closure. Access to crisis team programs was associated with significant changes in county-level drug overdose deaths, but not suicide or acute alcohol injury. Compared with counties with no change in access, crisis team program entry was associated with a 7% reduction in county-level drug overdose death rates, and crisis team program closure was associated with a 13% increase in drug overdose death rates. These findings may support the use of crisis teams as 1 intervention to address substance use disorder treatment gaps in the United States.

## Introduction

Behavioral health–related mortality (deaths from drug overdose, suicide, and acute alcohol injury) are leading causes of death in the United States, contributing to over 200 000 deaths in 2022.^1-6^ One response to this crisis was the replacement of the National Suicide Prevention line with a new behavioral health crisis line (988) in July 2022, marking a new era of investment in crisis services in the United States.^7^ The authorization of 988 spurred a series of state and federal initiatives intended to support crisis lines through expansion of mobile crisis teams and crisis stabilization services.^7,8^ In recognition that psychiatric comorbidity is common among populations with substance use disorders,^9^ crisis services now often include substance use disorder treatment—including detoxification, harm reduction, and outpatient services—in addition to the mental health treatment more commonly associated with crisis intervention.^10^

In particular, recent federal and state investments have focused on expanding access to crisis teams to improve crisis service availability in the community. Crisis teams have been operated by community mental health facilities since the 1980s and “provide rapid response, assess the individual, and resolve crisis situations.”^11,12^ Teams typically include a licensed clinician to assess the caller's behavioral health needs and connect the caller to a higher level of care, such as a crisis stabilization facility, emergency department (ED), or inpatient setting, if necessary.^13^ Historically, crisis teams have worked with crisis lines, including the National Suicide Prevention Lifeline and similar state-run crisis lines, as well as local law enforcement and other first responders in addition to the local specialty behavioral health system.^11^ As with other specialty behavioral health services, crisis teams were financed primarily via grant funding, although state Medicaid programs have increasingly played a role.^14^ Several observational matched-control studies have found that crisis teams were associated with reduced ED visits^15,16^ and psychiatric hospitalizations,^17^ while other studies noted cost savings from these programs,^18,19^ mostly due to reduced hospitalization.

An additional, but unmeasured, benefit of these programs may be reductions in behavioral health mortality. As a report from the National Association for State Mental Health Program Directors (NASMHPD) highlights, crisis teams may prevent behavioral health–related deaths through providing their clients new entry into a continuum of behavioral health crisis services.^20^ Crisis teams can offer their clients accessible, patient-centered services and intervene early during a crisis to connect clients with the evidence-based mental health and substance use treatment services that have been shown to reduce mortality, including peer support, medication, harm-reduction services, and other services and supports.^21^ Case studies in Oregon, Connecticut, San Francisco, and Arizona have described crisis teams’ capability to establish trust and rapport with clients in crisis—who are sometimes reluctant to seek care due to stigma—and link them to treatment and support services and divert them from incarceration or hospitalization.^13,22-24^ Better understanding the impact of crisis teams on mortality may help insurers and states assess coverage requirements and fee schedules: more than 25% of states did not cover mobile crisis services in fee-for-service Medicaid in 2022 and only a handful of states required commercial insurers cover mobile crisis team services.^14,25^

In this study, we examined whether changes in access to crisis teams embedded in mental health facilities are associated with changes in behavioral health mortality, making this the first national study to examine the association between crisis team interventions and health outcomes. Prior work has shown significant geographic variation in county-level access to crisis team services.^26,27^ We leverage this variation to assess whether entry and closure of crisis team programs were associated with changes in county-level behavioral health mortality—including drug overdose and alcohol injury in addition to suicide, in recognition that these programs serve individuals with substance use disorders. To better interpret any changes found, we considered whether effects differ when the change in access is due to the opening (or closing) of a mental health facility vs the introduction of a crisis team within an existing facility.

## Data and methods

### Study design

In this retrospective cohort study, we used county-level restricted Multiple Cause of Death files from the National Center for Health Statistics (2014–2019) paired with facility-level information obtained from the National Mental Health Treatment Directory. This study was exempted from review by the Yale Institutional Review Board, and no informed consent was required. We followed the Strengthening the Reporting of Observational Studies in Epidemiology (STROBE) guidelines for reporting observational studies.^28^

### Study sample

The study sample included all counties in the continental United States (*n* = 3110). Counties in Alaska and Hawaii were excluded because of changes in county designation over the study period.

### Exposures

Our exposure measures included county-level entry and closure of crisis team programs embedded in mental health facilities.

#### Identification of county-level crisis team access

We used the National Directory of Mental Health Treatment Facilities, which includes responses to the Substance Abuse and Mental Health Services Administration’s (SAMHSA's) National Mental Health Services Survey (N-MHSS), to identify facilities that offered crisis team services during our study period. The N-MHSS is fielded annually to all known mental health facilities, with response rates between 87% and 92% for the years studied. Responding facilities can choose to be included in the National Mental Health Treatment Directory, which identifies facilities by name and address in addition to reporting select N-MHSS responses. Approximately 75% of all mental health facilities choose to be included in the directory, making the directory the largest and most comprehensive census of mental health facilities. For each year included in the study period, the N-MHSS asked facilities to report whether they “offer a crisis intervention team that handles acute mental health issues at this facility and/or off-site” (yes or no). We used facility zip code to attribute facilities to counties and generate a binary outcome indicating the presence of at least 1 crisis team program for each county-year for 3110 US counties over 6 years (*n* = 18 666 county-years). The N-MHSS response rates and question text by year are included in Appendix Table A1.

#### Definition of county-level crisis team entry and closure

Using this county-year panel, we defined each county as either experiencing no change in crisis team access (either never had access or always had access), entry of a crisis team program (no program in 2014 and gained access to at least 1 program by 2019), or closure of a crisis team program (at least 1 program in 2014, and lost program access by 2019). In addition to Alaskan and Hawaiian counties excluded from the study sample, we excluded the 11% of continental US counties (*n* = 355) that gained and lost crisis team programs more than once during the study period.

Counties may have gained access to crisis teams either through the entry of a new mental health facility that offered crisis teams (in a county that may or may not have had a facility previously) or an existing facility newly adding crisis team services. Similarly, counties may have lost access to a crisis team through the closure of a program in an existing facility or through the closure of an entire facility. Thus, measured effects may be due to expanded (or reduced) treatment access. To consider whether our results were robust to counties that experienced changes only in crisis team program access (and not changes in facility access), in some specifications we limited our analysis to the 191 (39%) counties that experienced only crisis team program access changes (vs facility changes) in the year.

### Outcome measures

Our outcome measures were county-year death counts for suicide, drug overdose, and acute alcohol-related injury (death due to acute alcohol toxicity or withdrawal and excluding deaths from alcohol-related liver cirrhosis or motor vehicle accidents) identified using International Classification of Diseases, Tenth Revision (ICD-10), diagnoses (definitions shown in Appendix Table A2). We used Centers for Disease Control and Prevention (CDC) mortality definitions to attribute deaths, and accordingly outcomes were not mutually exclusive—that is, alcohol poisoning deaths were included in suicide and alcohol injury categories and drug poisoning deaths were included in drug overdose and suicide.

### Statistical analysis

We assessed standardized differences in baseline characteristics between counties experiencing crisis team program entry or closure and counties that did not experience any change in access (our comparison group) using a set of characteristics identified by prior literature to be associated with behavioral health mortality risk.^29-35^ We then examined county-level trends in suicide, drug overdose, and acute alcohol injury mortality rates during the study period (Appendix Figure A1). We next estimated the association between crisis team program access changes and behavioral health mortality using a county-year fixed-effects regression model similar to those used by prior studies estimating the impact of substance use treatment facility opening and closure on utilization and mortality^36,37^:


$$Outcome_{it}=\beta_{0}+\beta_{1}CrisisTeam_{it}+B_{2}Year_{t}+B_{3}County_{i}+\varepsilon_{it}$$

Where *i* denotes county, *t* denotes calendar year, and *Outcome_it_* was the number of county-year deaths from suicide, drug overdose, or acute alcohol injury (modeled separately*). CrisisTeam* was a time-varying binary variable that took on the value of 1 in the county-years that had a crisis team program, such that *β_1_* captured the aggregate difference in mortality rates after crisis team program entry or closure among the entry or closure (ie, treated) counties vs comparison counties. For each outcome we ran 2 regressions: first modeling entry (omitting closure counties from the sample), then closure (omitting entry counties). We included county fixed-effects to assess within-county changes in mortality associated with crisis team access and adjust for time-invariant county characteristics. We modeled the county-year death counts using Poisson distributions, which are robust to inference using panel data fixed-effects models.^38^ We included the log of each county's population as an offset to constrain the maximum count of deaths per county and used Huber-White robust standard errors. For ease of interpretation, we transformed model coefficients to incident rate ratios (IRRs; full results are shown in Appendix Tables A4 and A5). Because counties could gain or lose a crisis team program in any year of the study, we generated event study plots to examine trends in county-level mortality in the years before and after crisis team program entry and closure (Appendix Figure A2). Importantly, although our county-year fixed-effects model allowed us to control for time-invariant heterogeneity among the counties included in our cohort and isolate the association between crisis team program access and mortality, we cannot interpret a causal relationship between changes in crisis team program access and behavioral health mortality.

### Sensitivity analyses

We tested whether our main effects were robust to several different specifications, including negative binomial and linear models (Appendix Table A5). Additionally, consistent with new methodological recommendations, we also ran several models using the Callaway-Sant’anna estimator, which adjusts for potential differences in treatment effect by treatment cohort (eg, the year the crisis program entered or closed in a county) and changes in treatment effects over time.^39^ As an additional robustness check, we conducted a placebo test in which we randomized the year the crisis program entered or closed in a county (Appendix Table A7). Finally, because our study is observational, there may be unobserved differences between treatment and comparison counties that could bias our results. To address this concern, we constructed propensity scores from baseline covariates included in Table 1 and weighted regression models using inverse propensity score weights (Appendix Table A6).^40^ Analyses were performed using Stata version 14. *P* values <.05 were considered statistically significant.

**Table 1.** Baseline characteristics of US counties associated with changes in crisis team program access.

	No change in access, 2014–2019	Crisis team program entry, 2014–2019	Crisis team program closure, 2014–2019	Std. diff^a^	Std. diff^b^
No. of counties	2267	250	238		
Baseline county characteristics					
Mean county population	118 883	47 267	37 459	0.27	0.31
Demographic characteristics, mean %					
Below federal poverty line	16.6	17.0	16.9	−0.06	−0.04
Unemployed	8.4	8.8	8.4	−0.08	0.00
Without a high school diploma	15.0	15.1	15.2	−0.01	−0.03
Disabled	15.4	15.8	16.3	−0.09	−0.19
Age, mean %					
Age 65+ y	16.8	16.6	16.8	0.05	0.00
Age 17 y or younger	22.9	23.0	23.2	−0.04	−0.11
Race/ethnicity					
Non-White race, mean %	22.7	21.2	20.2	0.07	0.13
Social Vulnerability Index, mean	0.49	0.50	0.52	−0.03	−0.10
Uninsured , mean %	14.6	14.0	14.5	0.09	0.02
Rural, mean %	60.0	60.0	70.0	0.00	−0.27
Treatment capacity					
Mean no. of primary care providers per 10 000 population, 2015	5.2	5.5	4.7	−0.08	−0.08
Mean no. of psychiatrists per 10 000 population, 2015	0.4	0.3	0.2	0.13	0.13
Mean no. of hospital beds per 10 000 population, 2015	27.3	26.9	28.1	0.01	0.01

^a^Standardized difference (Std. diff) between counties experiencing crisis team entry (column 2) and counties with no change in access (column 1).

^b^Standardized difference (Std. diff) between counties experiencing crisis team closure (column 3) and counties with no change in access (column 1). A standardized difference greater than 0.10 or −0.10 is typically considered significant.

### Limitations

This study has several limitations. This study is observational, and so we can only report associations and are unable to conclude any causal relationship. Even though the N-MHSS directory is the largest known census of mental health facilities and has a consistently high response rate, survey nonresponse may have biased our estimates of county-level changes in access. In addition, the N-MHSS questionnaire in 2020 (the year after our study period ended) expanded the set of crisis team–related questions to ask facilities to whether they offer psychiatric crisis services “onsite” or, separately, “mobile/offsite.” While our findings should be interpreted in the context of the original survey question, the majority of facilities who reported offering crisis teams also reported offering on-/offsite psychiatric crisis services in 2020. Finally, this study was ecological: because the county was our unit of analysis (and we did not have patient-level data), we cannot say whether individuals in counties were treated by the crisis teams reported to be operating there. Counties also vary substantially in geographic size, and these differences may have contributed to differences in effect size.

## Results

### Characteristics of counties experiencing a change in crisis team program access

**Figure 1.**
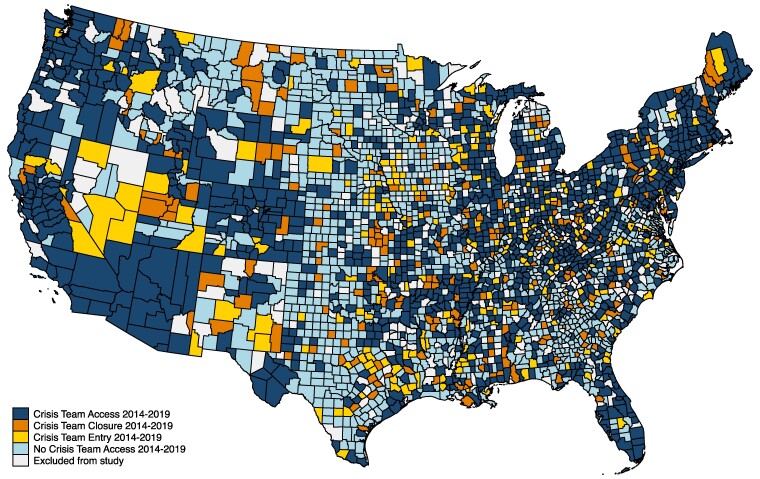
County-level variation in crisis team program entry and closure, 2014–2019. Sources: County-level crisis team program entry and closure was determined using responses to the National Mental Health Services Survey (N-MHSS) included in the National Directory of Mental Health Treatment Facilities. For each year included in the study period, the N-MHSS asked facilities to report whether they “offer a crisis intervention team that handles acute mental health issues at this facility and/or off-site” (yes or no). We defined each county as either experiencing no change in crisis team access (either never had access or always had access respectively), entry of a crisis team program (no program in 2014 and gained access to at least 1 program by 2019), or closure of a crisis team program (at least 1 program in 2014, and lost program access by 2019). In addition to Alaskan and Hawaiian counties excluded from the study sample, we excluded the 11% of continental US counties (*n* = 355) that gained and lost crisis team programs at more than 1 time point.

Among the 2755 counties included in the analysis, 488 (18%) experienced a change in access to crisis team programs from 2014 through 2019 (Figure 1). Approximately 9% of US counties experienced crisis team program entry (*n* = 250) during the study period, and approximately 9% experienced program closure (*n* = 238). Counties that experienced changes in access had smaller populations but were otherwise similar demographically to those that experienced no change in access (Table 1). Counties that experienced crisis team program closure were more likely to be rural than those experiencing program entry (70% vs 60%).

### Trends in county-level behavioral health mortality rates from 2014 through 2019

Our study included 558 897 decedents (273 119 to suicide, 271 493 to drug overdose, 14 285 to alcohol injury). Nationally, suicide and drug overdose mortality rates grew over the study period while mortality from acute alcohol injury decreased. At the county level, the percentage change in mortality rates over the study period varied significantly, where drug-overdose mortality rates had the largest variation in the percentage change over time (30-fold difference in county-level variation in percentage change in mortality rate over time, from −100% to 3002%; Appendix Figure A1).

### Associations of crisis team access change on behavioral health mortality

Crisis team program entry was associated with a 7% reduction in drug-overdose mortality rate among counties experiencing program entry compared with counties that had no change in access (IRR: 0.93; 95% CI: 0.86–1.00; Table 2). These findings were similar in magnitude in the subset of counties that experienced only crisis team program entry within an existing mental health treatment facility, rather than entry of a new facility (IRR: 0.93; 95% CI: 0.82–1.05), although the sample was much smaller and results were no longer statistically significant. Findings were robust to negative binomial, linear, and random-intercept specifications; inverse propensity-score weighting; and placebo testing (Appendix Tables A5–A7). Crisis team program entry was not associated with statistically significant changes in either suicide or acute alcohol injury.

**Table 2.** Changes in county-level behavioral health mortality rates associated with county-level crisis team program entry and closure.

		Associations with crisis team program entry			
Outcomes	Baseline mortality rate per 100 000 population	Counties experiencing any crisis team program entry (*n* = 250 counties)		Counties experiencing crisis team program entry with no new facility (*n* = 94 counties)	
		IRR	[95% CI]	IRR	[95% CI]
Suicide	18.55	1.03	[0.99–1.08]	1.06	[0.98-1.15]
Drug overdose	15.64	0.93	[0.86–1.00]**	0.93	[0.82–1.05]
Acute alcohol injury	0.85	0.85	[0.66–1.09]	0.66	[0.43–1.01]*

		Associations with crisis team program closure			
Outcomes	Baseline mortality rate per 100 000 population	All counties experiencing any crisis team program closure (*n* = 238 counties)		Counties experiencing crisis team program closure with no facility closure (*n* = 98 counties)	
Suicide	19.10	0.98	[0.94–1.03]	1.01	[0.92–1.10]
Drug overdose	13.65	1.13	[1.04–1.23]***	1.04	[0.94–1.15]
Acute alcohol injury	0.48	1.00	[0.804–1.24]	1.06	[0.75–1.50]

Main effects are from 6 separate Poisson regression county-year fixed-effects (2-way fixed-effects) models. The main effect is presented as an incident rate ratio (IRR), or the exponentiated coefficient of *CrisisTeam*, a binary variable that represents within-county effects of crisis team program entry or closure on mortality rates. Each model included an offset (the log of the county population) to constrain the total mortality count by the county population, effectively generating a rate. Therefore, the IRR can be interpreted as a percentage change in mortality rates, where values over 1.00 represent a percentage increase in the mortality rate (ie, 1.06 is a 6% increase in mortality rate) and values under 1.00 represent a decrease in mortality rate. All models were fit with Huber-White robust SEs. *P < .1, **P < .05, ***P < .001.

Conversely, crisis team program closure was associated with a 13% increase in drug-overdose mortality in the 4 years after closure relative to counties with no change in access (IRR: 1.13; 95% CI: 1.04–1.23; Table 2). This association was much smaller in magnitude and no longer statistically significant in the subset of counties that experienced only crisis team program closure vs facility closure. In contrast to the entry models, the increase in drug-overdose mortality was smaller in the subset of counties that experienced only crisis team program closure vs facility closure. Although the sample is small, we cannot say whether access to crisis team programs or the facilities in which these programs are located is the important factor influencing county-level drug-overdose mortality when facilities close. Main effects were robust to linear specification, inverse propensity-weighted analysis, and placebo testing, but were not robust to negative binomial specification. Crisis team program closure was not associated with statistically significant changes in suicide or acute alcohol injury. Findings from the event study (Appendix Figure A2) support the findings presented in Table 2.

## Discussion

In this retrospective cohort study of US counties from 2014 through 2019, we found that county-level access to crisis teams was associated with changes in county-level drug-overdose mortality but not suicide or alcohol injury. This study is the first national study to examine the association between crisis team interventions and health outcomes. Most prior studies evaluating crisis teams and similar crisis services have focused on utilization outcomes, such as psychiatric hospitalization or ED use, and have typically been single-center or single-state studies. A recent study by Burns and colleagues^16^ found that access to crisis services (at the zip-code level) in 5 states was associated with reductions in behavioral health–related ED visit rate. Our findings that crisis team program access was associated with reduced drug-overdose mortality rates complement Burns et al’s findings of reduced ED utilization as well as recent case reports documenting crisis teams’ roles in providing new access to substance use disorder treatment for their clients.

Importantly, we did not detect any difference in county-level suicide rate associated with changes in crisis team access. Our findings complement null findings from Mark and colleagues’ recently published study^41^ evaluating Arizona's model comprehensive crisis care program, which implemented crisis lines, crisis teams, and crisis stabilization centers, and found that crisis service implementation was associated with only small relative reductions in suicide-related hospitalization trends in Arizona compared with a neighboring state. Our findings do not contradict the impact of crisis teams in other important, more proximal, outcomes, such as ED visits or criminal justice-diversion. Instead, they suggest the need for future studies to assess the impact of crisis teams on suicide prevention specifically, including how and whether crisis teams work to engage clients in community-based mental health services post-crisis, safety planning, and involuntary commitment procedures.

Our findings instead underscore the potential benefit of crisis teams in addressing gaps in access to substance use disorder treatment. Indeed, our finding that crisis team entry was associated with a reduction in drug overdose deaths complements findings from prior studies that found that substance use treatment facility entry was associated with a reduction in drug-related ED visits^36^ and county-level drug overdose mortality rates.^37^ The role of crisis teams in substance use disorder treatment has historically been overlooked. Yet, co-occurring substance use and mental health disorders are common,^9^ adults in mental distress are at increased risk of drug overdose,^42^ and a recent cohort study of over 9000 crisis service encounters in Arizona found that 54% of crisis service episodes had a documented substance use disorder.^43^ Recent case studies and revised practice guidelines have described the role that crisis teams play in connecting patients to substance use treatment and overcoming traditional access barriers related to cost or lack of motivation to stop using.^22,23^ These reports have described how crisis teams engage clients in substance use disorder services that may prevent drug-related overdose,^44^ including harm-reduction services in addition to detoxification services, medication, and other outpatient treatment.^45-47^ Delayed access to addiction treatment increases the risk of overdose and other adverse outcomes.^48,49^ Yet, insufficient program capacity, wait times, and payment barriers can prevent rapid access, and inadequate support during transitions in the care continuum, such as after ED and hospital discharge, can leave patients vulnerable.^50,51^ Crisis teams may help address well-documented gaps in the care continuum for immediate services similarly to bridge clinics, which offer same- and next-day low-barrier access to services, stabilization, and linkage to long-term care, and show promising early evidence of facilitating treatment initiation and retention.^52^ Because crisis teams can include peers, crisis teams may refer patients to other peer-led crisis support services,^53^ such as peer respite or “living rooms,” a community-based, peer-run alternative to hospitalization^54^ that may be more likely to engage patients who otherwise would not be interested or not be admitted to treatment programs. In addition, most crisis services are funded or supported by state-funded programs intended to be free or low cost, overcoming cost barriers associated with substance use disorder treatment.^25^

We also did not detect any difference in county-level acute alcohol injury associated with changes in crisis team access. Although we would hypothesize that crisis teams could be as effective in referring patients to alcohol use disorder (AUD) treatment as other substance use disorders, specialty behavioral health facilities are less likely to offer AUD treatment than other substance use disorders, such as treatment for opioid use disorder,^55^ which could prevent effective referral from crisis teams. In addition, there were fewer acute alcohol injury–related deaths at the county level, limiting our statistical power.

Taken together, findings from this study suggest that access to crisis teams is associated with reductions in drug-overdose deaths and underscores the importance of harnessing new payment and financing methods to sustain and increase access to crisis response. For example, states could consider using opioid settlement funds^56^—especially funds allocated to counties that are often the entities managing local behavioral health crisis services—to fund new crisis teams. States can also leverage existing parity laws to ensure that health plans cover crisis care services at parity with emergency medical services. There are also multiple Medicaid financing mechanisms that could increase crisis service supply. These include state planning grants focused on crisis system development, enhanced federal Medicaid matching rates for qualifying mobile crisis services, and the Certified Community Behavioral Health Clinic Program (CCBHC), a Medicaid demonstration that was expanded to 10 additional states in 2022 via the Bipartisan Safer Communities Act.^25,57,58^ The CCBHC demonstration clinics in participating states receive prospective Medicaid payment^59^ at enhanced rates relative to other outpatient clinics and, in return, are required to offer comprehensive behavioral health services, including crisis care.^60^

## Conclusion

In this national retrospective cohort study, we found that entry of crisis teams into a county was associated with a reduction in county-level overdose mortality, while the closure of crisis teams was associated with an increase in overdose mortality. Changes in crisis team access had limited observed effects on county-level suicide or acute alcohol injury mortality.

## Supplementary Material

qxaf003_Supplementary_Data
