# Supplementary material for: Is access to crisis teams associated with changes in behavioral health mortality?
Source: Health Aff Sch. 2025 Jan 15;3(1):qxaf003. doi: 10.1093/haschl/qxaf003 (PMC11772998; doi:10.1093/haschl/qxaf003)
Supplement: qxaf003_Supplementary_Data [file qxaf003_supplementary_data.zip › Crisis Teams_HAS_Appendix_01022025.docx]

**Is access to crisis teams associated with changes in behavioral health mortality?**

**Technical Appendix:**

**Appendix Table A1:** N-MHSS questions, by survey year and N-MHSS response rate

**Appendix Table A2:** ICD-10 definitions from Mortality Outcome ICD-10 Definitions

**Appendix Figure A1:** Percent change in county-level age-adjusted behavioral health mortality rates 2014- 2019

**Appendix Table A3:** County-year fixed effect Poisson model regression output (Entry models)

**Appendix Table A4:** County-year fixed effect Poisson model regression output (Closure models)

**Appendix Figure A2:** Associations between county-level crisis team access changes and behavioral health mortality over time (Event study plots)

**Appendix Table A5:** Sensitivity analysis comparing Poisson with negative binomial and linear specifications

**Appendix Figure A3:** Propensity score distribution among entry/closure and comparison counties

**Appendix Table A6:** Sensitivity analysis using inverse propensity score weights (IPW)

**Appendix Table A7:** Robustness check using placebo entry and closure dates

| **Appendix Table A1:** N-MHSS CIT questions, by survey year and N-MHSS response rate | | | |
| --- | --- | --- | --- |
| Year | N-MHSS question | N-MHSS unit response rate | N-MHSS item response rate |
| [2014](https://www.datafiles.samhsa.gov/sites/default/files/field-uploads-protected/studies/N-MHSS-2014/N-MHSS-2014-datasets/N-MHSS-2014-DS0001/N-MHSS-2014-DS0001-info/N-MHSS-2014-DS0001-info-questionnaire-specs.pdf) | “Does this facility offer a crisis intervention team that handles acute mental health issues at this facility and/or off-site” (yes/no) | 88.1 % | 96.9% |
| [2015](https://www.datafiles.samhsa.gov/sites/default/files/field-uploads-protected/studies/N-MHSS-2015/N-MHSS-2015-datasets/N-MHSS-2015-DS0001/N-MHSS-2015-DS0001-info/N-MHSS-2015-DS0001-info-questionnaire-specs.pdf) | “Does this facility offer a crisis intervention team that handles acute mental health issues at this facility and/or off-site” (yes/no) | 91.9% | 97.9% |
| [2016](https://www.datafiles.samhsa.gov/sites/default/files/field-uploads-protected/studies/N-MHSS-2016/N-MHSS-2016-datasets/N-MHSS-2016-DS0001/N-MHSS-2016-DS0001-info/N-MHSS-2016-DS0001-info-questionnaire-specs.pdf) | “Does this facility offer a crisis intervention team that handles acute mental health issues at this facility and/or off-site” (yes/no) | 91.1% | 97.6% |
| 2017 | “Does this facility offer a crisis intervention team that handles acute mental health issues at this facility and/or off-site” (yes/no) | 87% | 98% |
| [2018](https://www.samhsa.gov/data/sites/default/files/cbhsq-reports/NMHSS-2018.pdf) | “Does this facility offer a crisis intervention team that handles acute mental health issues at this facility and/or off-site?” (yes/no) | 90% | 99% |
| [2019](https://www.samhsa.gov/data/sites/default/files/reports/rpt29388/2019_NMHSS/2019-NMHSS-R.pdf) | “Does this facility offer a crisis intervention team that handles acute mental health issues at this facility and/or off-site?” (yes/no) | 91% | 99% |
| The source questionnaires (available on SAMHSA’s website) are hyperlinked for each year. The N-MHSS questionnaire in 2020 (the year after our study ended) did expand the set of crisis team-related questions to ask facilities to distinguish between offering psychiatric crisis services “onsite” or, separately, “mobile/offsite” in addition to asking about crisis intervention teams. In a separate analysis using 2020 data, we found that most facilities (75% and 87% respectively) who reported offering psychiatric crisis services onsite or mobile/offsite also reported offering crisis intervention teams. | | | |

| **Appendix Table A2:** Mortality Outcome ICD-10 Definitions | |
| --- | --- |
| Mortality Outcome | ICD-10 Diagnosis Definition(underlying cause of death) |
| Suicide^a^ | U03, X61-X85, Y87 |
| Drug overdose^b^ | X40-44, X60-64, X85, and Y10-Y14 |
| Acute alcohol injury^c^* | E24.4, F10, G31.2, G62.1, G72.1, K29.2 K86.0, R78.0, X45, X65, Y15 |
| **Notes:** Data source is National Center for Health Statistics (NCHS) National Vital Statistics System (restricted-use multiple cause of death file).  a = Suicide [Mortality](https://www.cdc.gov/nchs/products/databriefs/db330.htm) in the United States, 1999–2017  b = [Trends and Geographic Patterns in Drug and Synthetic Opioid Overdose Deaths — United States, 2013–2019](https://www.cdc.gov/mmwr/volumes/70/wr/mm7006a4.htm?s_cid=mm7006a4_w),  c = [Alcohol-induced Death Rates in the United States, 2019–2020](https://www.cdc.gov/nchs/products/databriefs/db448.htm#:~:text=Vital%20Statistics%20System-,Rates%20of%20alcohol%2Dinduced%20deaths%20generally%20increased%20from%202000%20to,in%202020%20(Figure%201).)  (*alcohol injury definition excludes alcoholic liver disease (K70), alcohol-induced acute pancreatitis (K85.2), and alcoholic cardiomyopathy (I42.6)) | |

**Appendix Figure A1:** Percent change in county-level age-adjusted behavioral health mortality rates 2014- 2019

**Appendix Figure A1a:** Percent change in age-adjusted suicide mortality rate per 100,000 population, 2014-2015 to 2018-2019


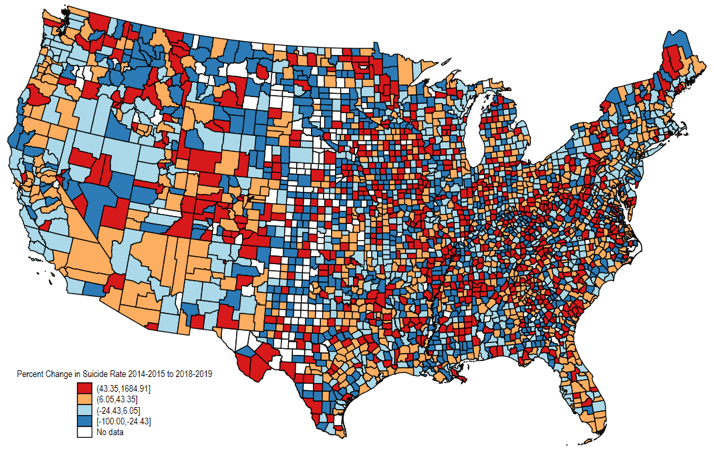


**Appendix Figure A1b:** Percent change in age-adjusted overdose mortality rate per 100,000 population, 2014-2015 to 2018-2019


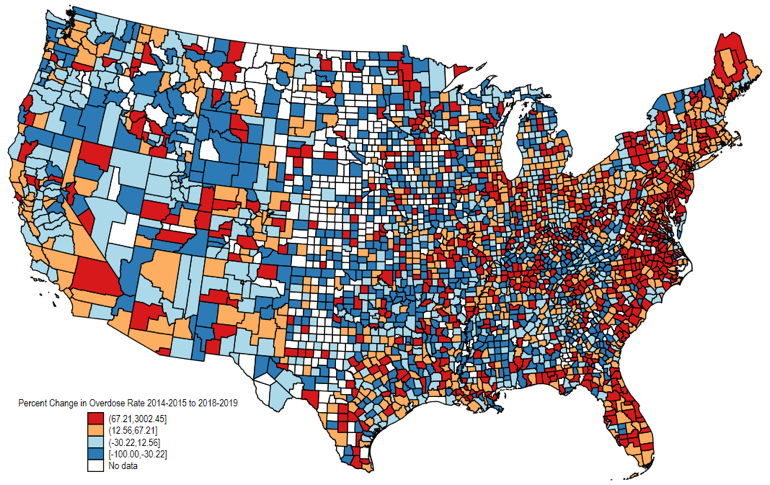


**Appendix Figure A1c:** Percent change in age-adjusted alcohol injury mortality rate per 100,000 population, 2014-2015 to 2018-2019


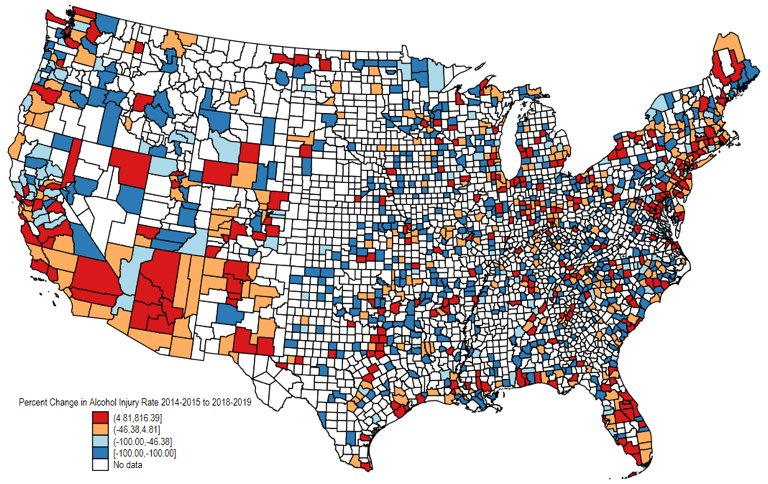


| **Appendix Table A3:** County-year fixed effect Poisson model regression output, by outcome (Entry models) | | | |
| --- | --- | --- | --- |
| **Treatment:** Counties experiencing crisis team program entry (vs. counties with no change in access) |  |  |  |
| VARIABLES | Dep Var: Suicide | Dep Var: Drug Overdose | Dep Var: Acute Alcohol Injury |
|  |  |  |  |
| 1.any_crisisteam | 1.033 | 0.928** | 0.850 |
|  | (0.988 - 1.081) | (0.863 - 0.999) | (0.660 - 1.094) |
| 2015.year | 1.028*** | 1.121*** | 1.003 |
|  | (1.013 - 1.042) | (1.100 - 1.143) | (0.942 - 1.069) |
| 2016.year | 1.041*** | 1.377*** | 1.042 |
|  | (1.026 - 1.057) | (1.329 - 1.427) | (0.963 - 1.127) |
| 2017.year | 1.098*** | 1.523*** | 1.017 |
|  | (1.081 - 1.115) | (1.465 - 1.582) | (0.949 - 1.090) |
| 2018.year | 1.127*** | 1.460*** | 0.985 |
|  | (1.110 - 1.144) | (1.408 - 1.514) | (0.922 - 1.053) |
| 2019.year | 1.105*** | 1.531*** | 1.000 |
|  | (1.088 - 1.122) | (1.473 - 1.591) | (0.919 - 1.089) |
| Constant |  |  |  |
|  |  |  |  |
|  |  |  |  |
| Observations | 14,946 | 14,148 | 8,832 |
| Number of numeric_fips | 2,491 | 2,358 | 1,472 |
| *** p<0.01, ** p<0.05, * p<0.1  Note: all coefficients are represented as incident rate ratios (IRR) for ease of interpretation. All models include county fixed effects, which are not shown here, in addition to the calendar year fixed effects, which are shown here. Counties without any deaths during the study period were excluded from the models: for suicide models, 25 counties were excluded (1 entry (0.4%) , 24 comparison (1.1%)), for overdose models, 158 counties were excluded (4 entry (1.6%), 154 comparison (6.8%)), and for alcohol injury models, 1,044 counties were excluded (93 entry (37.2%) and 951 comparison (42.0%)). | | | |

| **Appendix Table A4:** County-year Poisson model regression output, by outcome (Closure models) | | | |
| --- | --- | --- | --- |
| **Treatment:** Counties experiencing crisis team program closure (vs. counties with no change in access) |  |  |  |
| VARIABLES | Dep Var: Suicide | Dep Var: Drug Overdose | Dep Var: Acute Alcohol Injury |
|  |  |  |  |
| 1.any_crisisteam | 0.979 | 1.130*** | 0.998 |
|  | (0.935 - 1.026) | (1.037 - 1.231) | (0.804 - 1.238) |
| 2015.year | 1.027*** | 1.123*** | 1.006 |
|  | (1.012 - 1.042) | (1.102 - 1.145) | (0.944 - 1.072) |
| 2016.year | 1.043*** | 1.373*** | 1.050 |
|  | (1.027 - 1.059) | (1.325 - 1.423) | (0.971 - 1.137) |
| 2017.year | 1.098*** | 1.520*** | 1.009 |
|  | (1.081 - 1.116) | (1.462 - 1.580) | (0.941 - 1.082) |
| 2018.year | 1.129*** | 1.463*** | 1.000 |
|  | (1.113 - 1.146) | (1.411 - 1.517) | (0.936 - 1.069) |
| 2019.year | 1.104*** | 1.534*** | 1.003 |
|  | (1.086 - 1.121) | (1.476 - 1.594) | (0.921 - 1.093) |
| Constant |  |  |  |
|  |  |  |  |
|  |  |  |  |
| Observations | 14,874 | 14,052 | 8,664 |
| Number of numeric_fips | 2,479 | 2,342 | 1,444 |
| *** p<0.01, ** p<0.05, * p<0.1  Note: all coefficients are represented as incident rate ratios (IRR) for ease of interpretation. All models include county fixed effects, which are not shown here, in addition to the calendar year fixed effects, which are shown here. Counties without any deaths during the study period were excluded from the models: for suicide models, 24 counties were excluded (0 closure (0.0%), 24 comparison (1.1%)), for overdose models, 161 counties were excluded (7 closure (2.9%), 154 comparison (6.8%)), and for alcohol injury models, 1,059 counties were excluded (108 closure (45.6%) and 951 comparison (42.0%)). | | | |

**Appendix Figure A2:** Associations between county-level crisis team access changes and behavioral health mortality over time

Entry

Closure


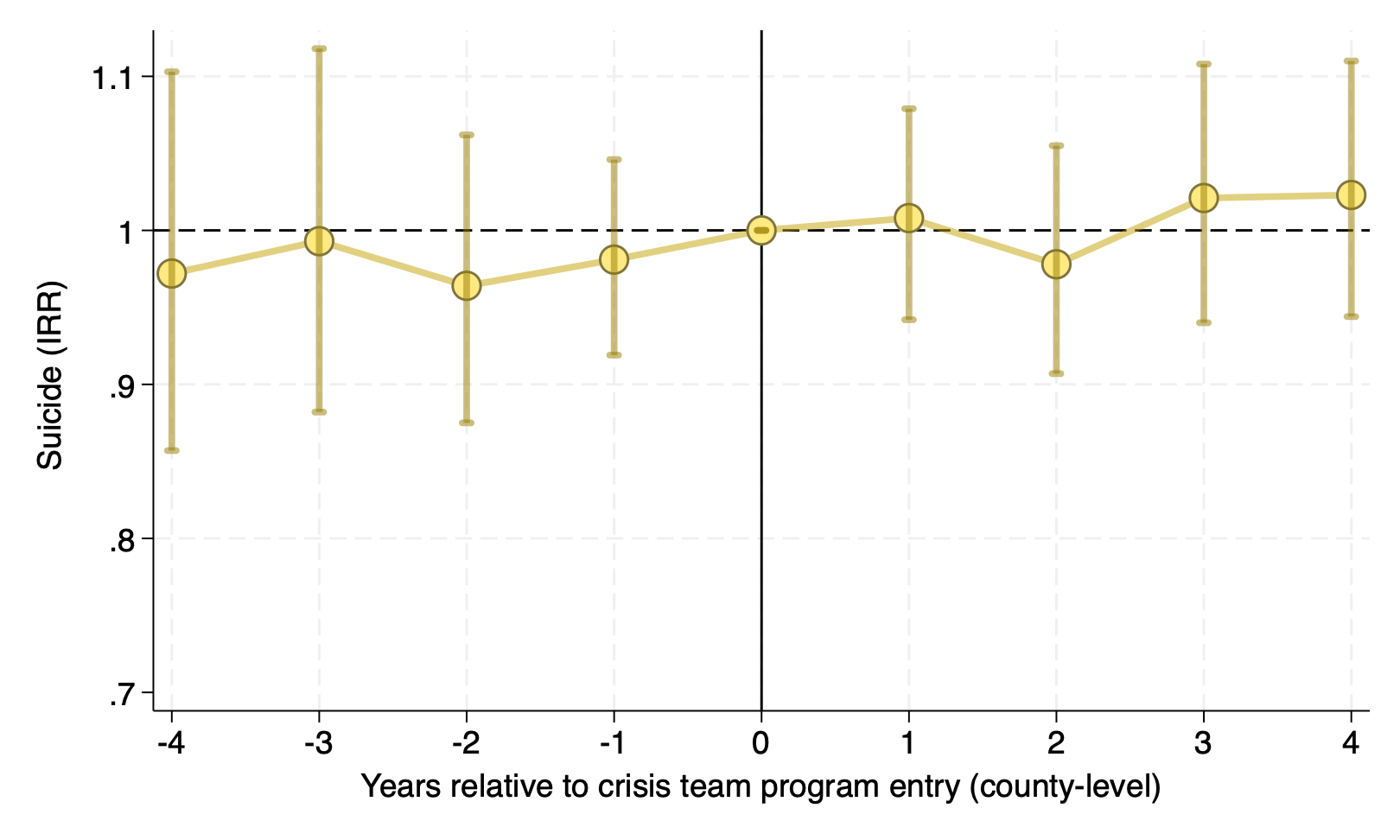

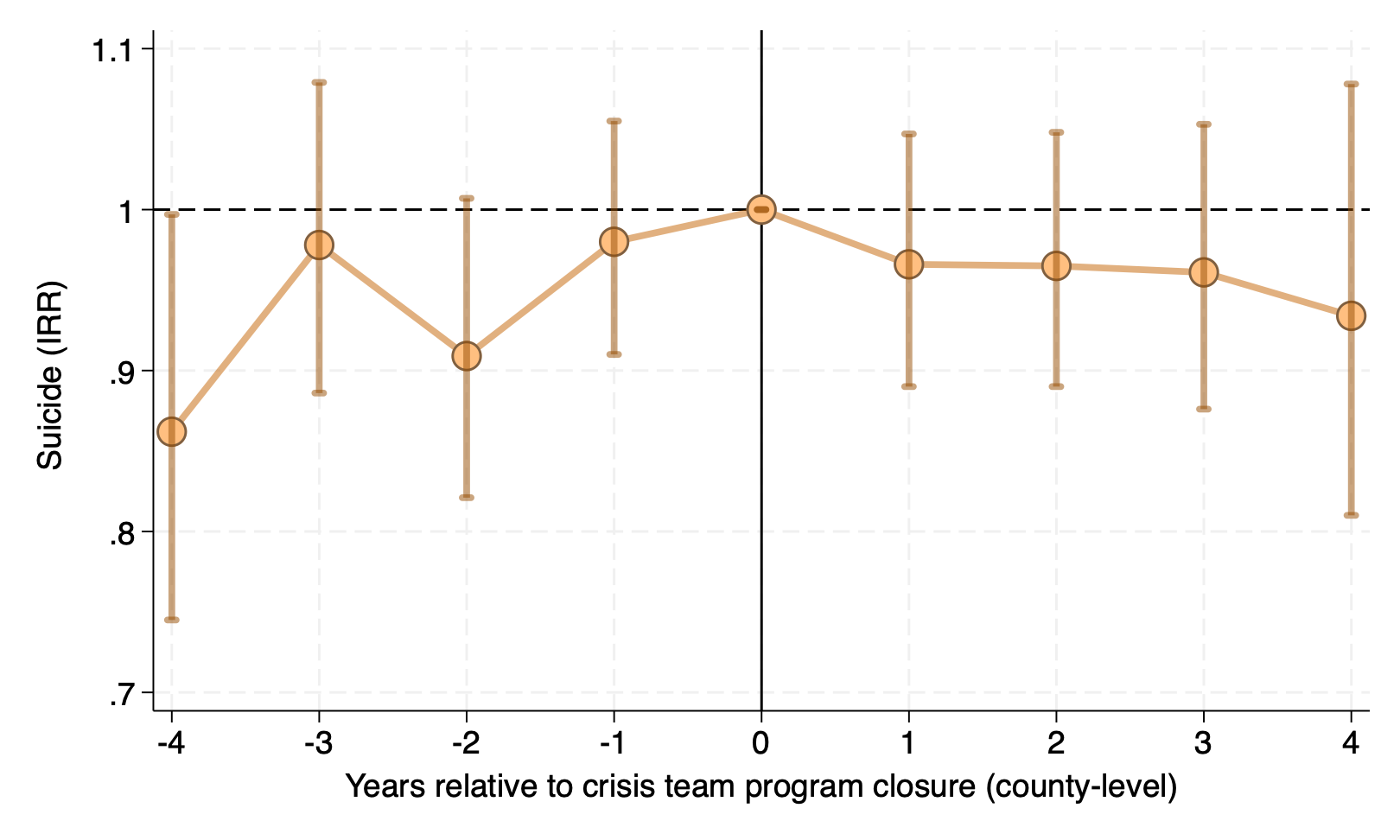


2a. Suicide


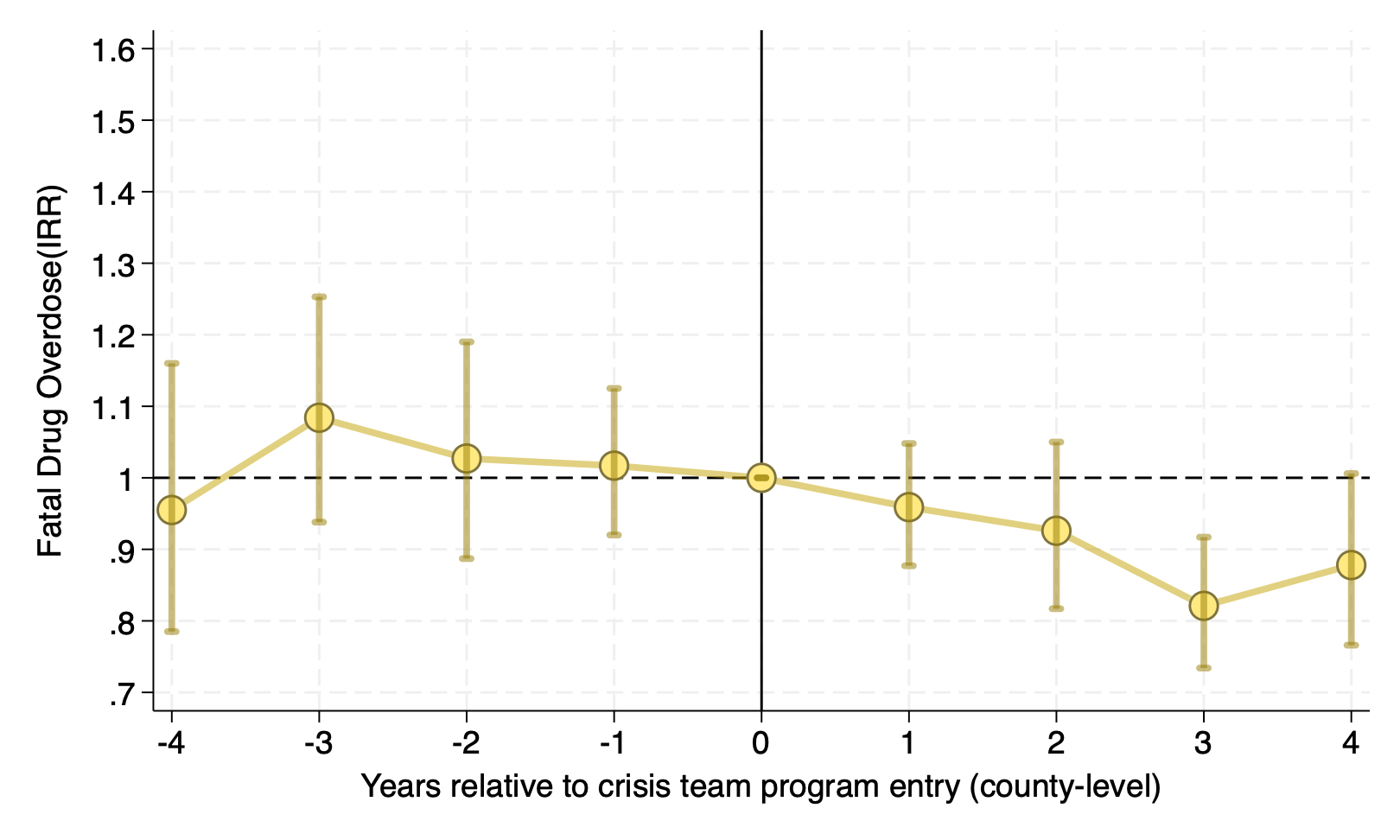

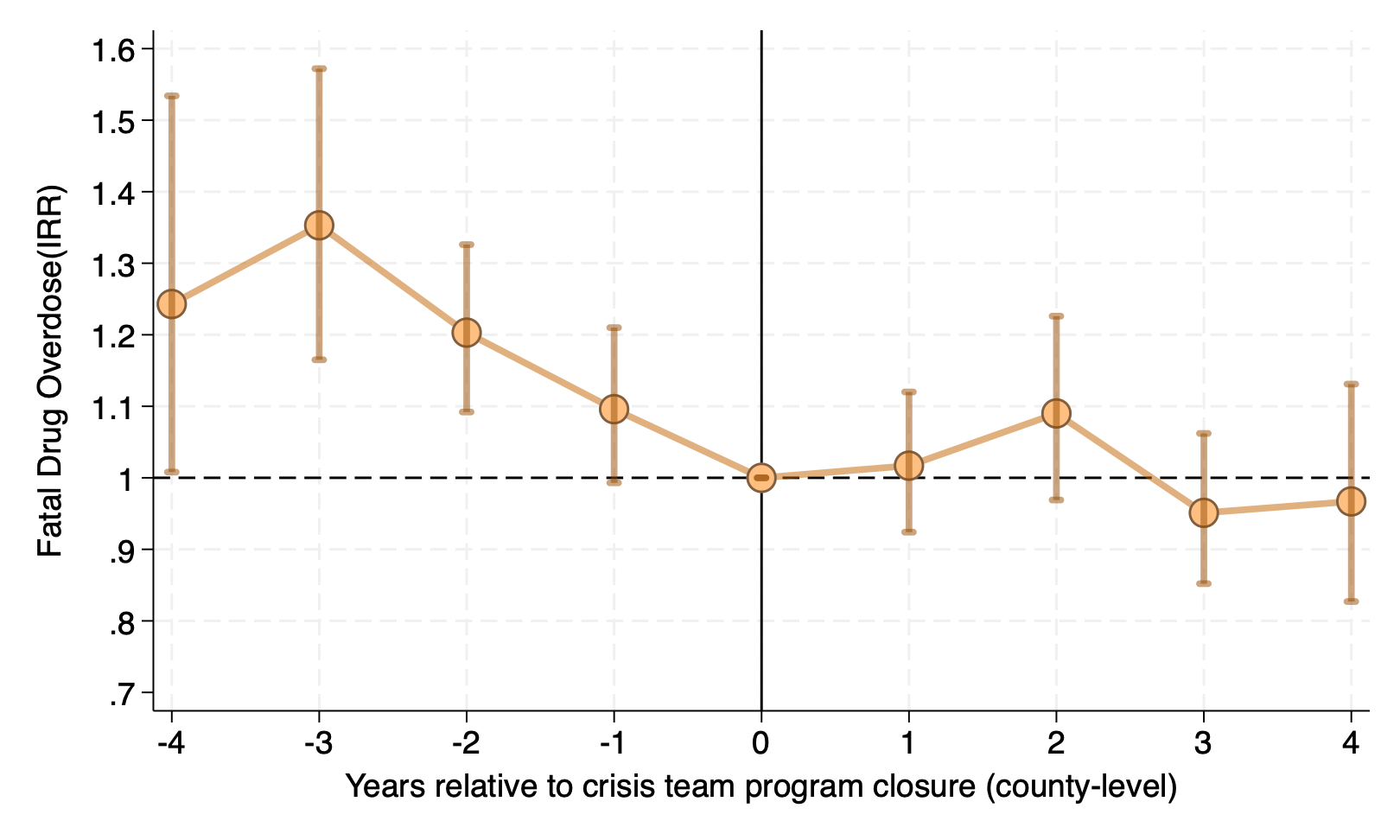


2b. Drug Overdose


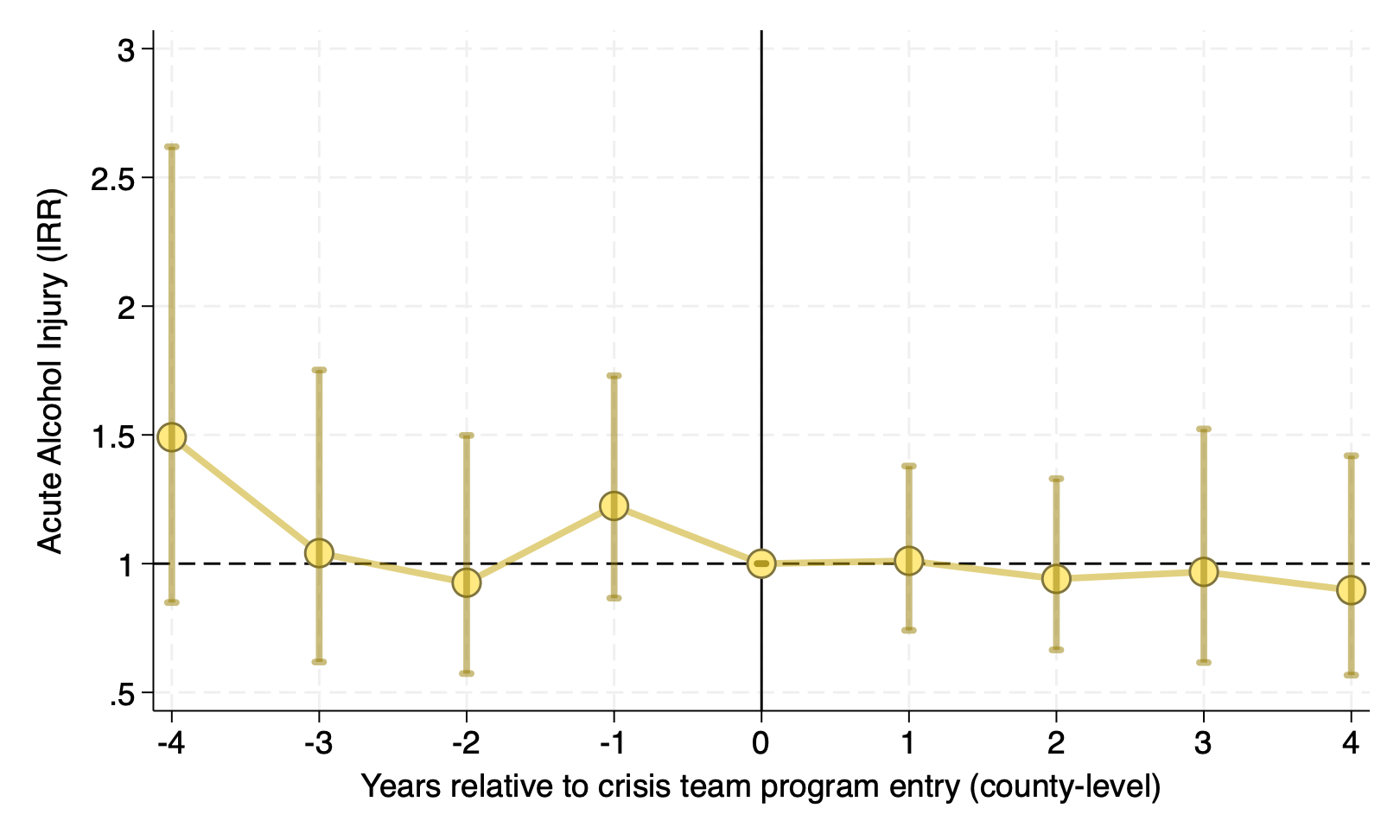

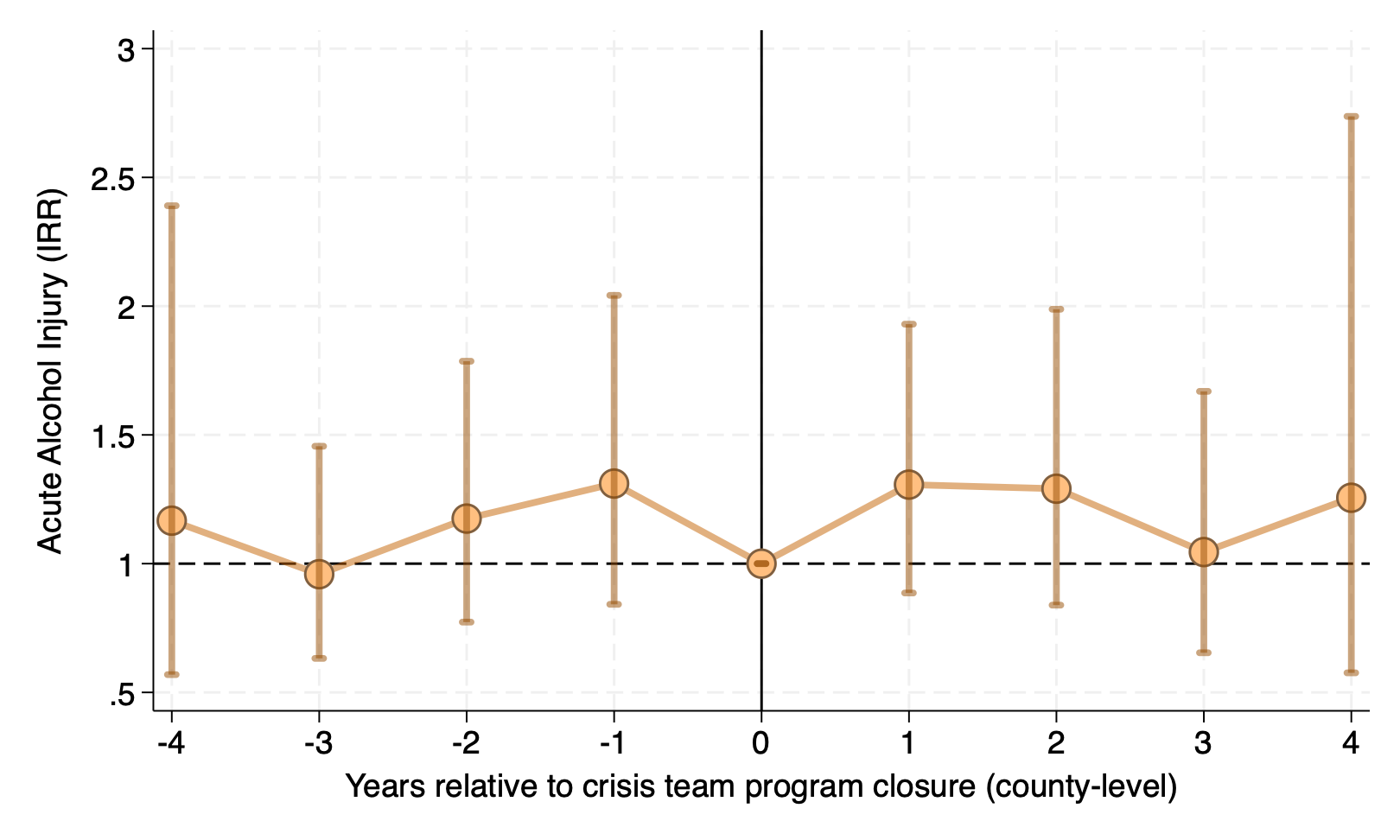


2c. Acute Alcohol Injury

**Note**: IRR = incident rate ratio. Estimates plotted in figures 2a-2c come from six separate event-study models, which compared the relative change in behavioral mortality rates among the counties that experienced crisis team program entry relative to counties with no change in access for the years before and after the change year (year=0), and rates among counties that experience crisis team program closure relative to counties with no change in access. All event study models included county-year fixed effects, modeled outcomes using Poisson distribution, and were fit with Huber-White robust standard errors.

**Appendix Table A5:** Sensitivity analysis comparing coefficients across multiple specifications

|  | Baseline rate per 100,000 | Poisson  TWFE | | Negative Binomial  TWFE | | Linear  TWFE | | Linear (CS) | | Poisson random-intercept model | |
| --- | --- | --- | --- | --- | --- | --- | --- | --- | --- | --- | --- |
| **Entry** |  | coef | [95% CI] | coef | [95% CI] | coef | [95% CI] | coef | [95% CI] | coef | [95% CI] |
| Suicide | 18.55 | 1.03 | [0.99-1.08] | 1.03 | [0.99-1.08] | 1.03 | [0.96-1.10] | 1.00 | [0.84-1.20] | 1.04 | [0.88-1.23] |
| Drug Overdose | 15.64 | 0.93** | [0.86-1.00] | 0.79*** | [0.75-0.84] | 0.95 | [0.89-1.02] | 0.83 | [0.66-1.04] | 0.60*** | [0.44-0.83] |
| Alcohol Injury | 0.85 | 0.85 | [0.66-1.09] | 0.81* | [0.65-1.01] | 0.95 | [0.82-1.11] | 0.49 | [0.04-5.98] | 0.66 | [0.31-1.41] |
| **Closure** |  |  |  |  |  |  |  |  |  |  |  |
| Suicide | 19.10 | 0.98 | [0.94-1.03] | 0.98 | [0.93-1.03] | 0.97 | [0.91-1.03] | 1.05 | [0.92-1.20] | 0.89 | [0.76-1.18] |
| Drug Overdose | 13.65 | 1.13*** | [1.04-1.23] | 0.92*** | [0.86-0.97] | 1.07* | [1.00-1.15] | 0.95 | [0.83-1.08] | 1.03 | [0.74-1.44] |
| Alcohol Injury | 0.48 | 1.00 | [0.804-1.24] | 0.93 | [0.73-1.18] | 1.00 | [0.32-1.13] | 0.98 | [0.79-1.21] | 0.71 | [0.24-2.10] |

*** p<0.01, ** p<0.05, * p<0.1

**Note:** Values in eTable5 come from 24 separate regression models to compare main effects from our primary specification (Poisson TWFE) with estimates from 3 other specifications (Negative Binomial TWFE, Linear TWFE, and Linear Callaway Sant’anna (CS)) for each outcome (suicide, drug overdose, acute alcohol injury) for entry and, separately, closure counties, relative to counties with no change in access. All fixed effect models included county and year fixed effects. For Poisson and Negative Binomial models, outcomes were county-year death counts, whereas for the linear models, outcomes were modeled as the log of the mortality rate per 100,000 population. Accordingly, all values in table are exponentiated for ease of interpretation. Poisson random-intercept model included numerous county and state-level control variables in addition to year fixed effects, including: county geographic size (square miles), population size, whether or not the county was designated rural (RUCA >=4), the percent of the county population living at or below the federal poverty limit, unemployed, without a high school diploma, age >65, age <17, disabled, single parent, part of racial/ethnic minority group, uninsured. This model also included a number of county-level treatment capacity variables including the number of primary care clinicians per 10,000 population, hospital beds per 10,000 population, as well as multiple state laws that could influence crisis team entry or closure, including gun laws (e.g., waiting period laws and child access laws), naloxone prescribing authority, Medicaid expansion, as well as whether the state had participated in the Certified Behavioral Health Clinic (CCBHC) demonstration.

**Appendix Figure A3:** Distribution of propensity scores for crisis team program entry and closure

**Appendix Figure A3a:** Probability of crisis team program entry among counties that experienced program entry (1) vs. no entry (0)


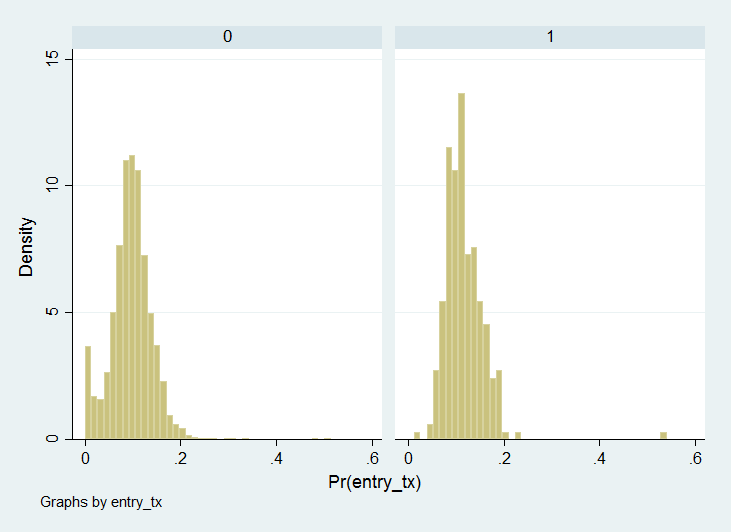


**Appendix Figure A3b:** Probability of crisis team program closure among counties that experienced program closure (1) vs. no closure (0)


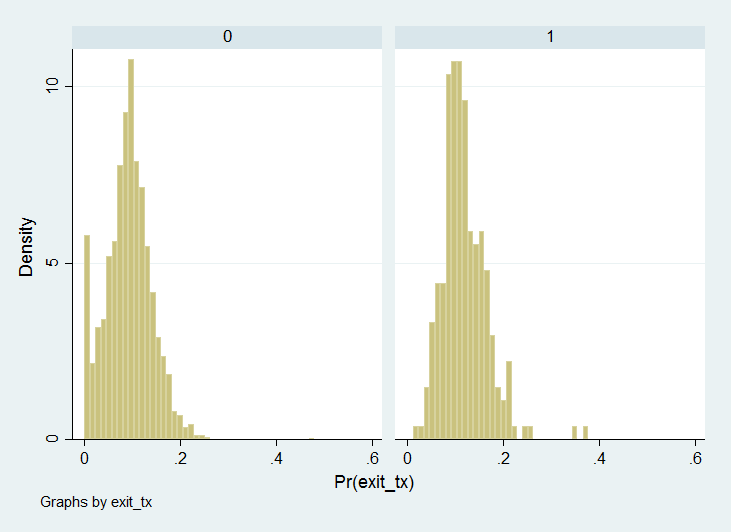


**Note:** eFigure 3a and b show the probability distribution from two logit regression models that estimate the probability of crisis team program entry (or closure, separately) as a function of all baseline county covariates included in Table 1. The similarly in the distribution and spread of both histograms demonstrates common support, or overlap, between the potential outcomes of the treatment and comparison groups.

**Appendix Table A6:** Sensitivity analysis using inverse propensity score weights (IPW)

| **Entry** | Baseline rate per 100,000 | Unweighted (main specification) |  | IPW-weighted |  |
| --- | --- | --- | --- | --- | --- |
|  |  | IRR | [95% CI] | IRR | [95% CI] |
| Suicide | 18.55 | 1.03 | [0.99-1.08] | 1.05 | [1.04-1.05]*** |
| Drug Overdose | 15.64 | 0.93** | [0.86-1.00] | 0.97 | [0.95-0.99]** |
| Acute Alcohol Injury | 0.85 | 0.85 | [0.66-1.09] | 0.84 | [0.80-0.87]*** |

| **Closure** | Baseline rate per 100,000 | Unweighted (main specification) |  | IPW-weighted |  |
| --- | --- | --- | --- | --- | --- |
|  |  | IRR | [95% CI] | IRR | [95% CI] |
| Suicide | 19.10 | 0.98 | [0.94-1.03] | 0.97 | [0.96-0.98]*** |
| Drug Overdose | 13.65 | 1.13*** | [1.04-1.23] | 1.15 | [1.12-1.17]*** |
| Acute Alcohol Injury | 0.48 | 1.00 | [0.804-1.24] | 0.97 | [0.93-1.02] |

*** p<0.01, ** p<0.05, * p<0.1

**Appendix Table A7:** Robustness check using placebo entry and closure dates

|  | Placebo entry/closure | Distribution of beta coefficient across 1000 simulations | | | | | | | |
| --- | --- | --- | --- | --- | --- | --- | --- | --- | --- |
|  | Mean beta coefficient | P2.5 | P10 | P25 | P50 | P75 | P90 | P97.5 | |
| **Entry** |  |  |  |  |  |  |  |  | |
| Suicide | 1.001 | 0.961 | 0.974 | 0.988 | 1.002 | 1.014 | 1.028 | 1.042 | |
| Drug Overdose | 1.001 | 0.946 | 0.965 | 0.983 | 1.000 | 1.021 | 1.038 | 1.059 | |
| Alcohol Injury | 1.005 | 0.839 | 0.905 | 0.945 | 1.007 | 1.072 | 1.122 | 1.203 | |
| **Closure** |  |  |  |  |  |  |  |  | |
| Suicide | 0.999 | 0.953 | 0.968 | 0.982 | 0.999 | 1.015 | 1.030 | 1.047 | |
| Drug Overdose | 0.999 | 0.935 | 0.958 | 0.975 | 0.998 | 1.022 | 1.043 | 1.067 | |
| Alcohol Injury | 1.010 | 0.799 | 0.865 | 0.926 | 1.008 | 1.096 | 1.179 | 1.277 | |
| Note: We conducted a placebo test where we randomly generated placebo entry and closure dates (using Stata user-written command *shufflevar*) within our treatment groups and replicated our main specification (county-year fixed effect Poisson models) 1000 times using Monte Carlo simulations (using Stata user-written command *simulate*) as an additional robustness check. This table shows the distribution of the simulated treatment effect using placebo entry/closure dates (beta coefficient) and its distribution. | | | | | | | | |  |
